# Na^+^/H^+^ Exchanger 1 Inhibition Overcomes Venetoclax Resistance in Acute Myeloid Leukemia

**DOI:** 10.3390/cells14221759

**Published:** 2025-11-10

**Authors:** Shin Young Hyun, Eun Jung Na, Yu Ri Kim, Yoo Hong Min, June-Won Cheong

**Affiliations:** 1 Department of Internal Medicine, Yonsei University College of Medicine, Seoul 03722, Republic of Korea; syhyun@yuhs.ac (S.Y.H.);; 2 Blood Cancer Research Institute, Yonsei University College of Medicine, Seoul 03722, Republic of Korea; 3 College of Pharmacy and Graduate School of Pharmaceutical Sciences, Ewha Womans University, Seoul 03760, Republic of Korea; yardpie@naver.com

**Keywords:** acute myeloid leukemia, Na-H exchanger 1, venetoclax, PI3K/Akt pathway, resistance

## Abstract

Despite advances with novel targeted agents (e.g., BCL-2 or IDH inhibitors) combined with chemotherapy for acute myeloid leukemia (AML), drug resistance persists. We investigated whether blocking Na^+^/H^+^ exchanger 1 (NHE1) could enhance AML cell sensitivity to the BCL-2 inhibitor venetoclax and sought to determine the molecular mechanisms. Our results demonstrated that co-treatment with venetoclax and the NHE1 inhibitor 5-(N,N-hexamethylene) amiloride (HMA) synergistically induced apoptosis in both venetoclax-sensitive and -resistant leukemic cell lines. Specifically, the combination significantly increased apoptosis in venetoclax-resistant THP-1 cells to 72.28% (17.79% with 100 nM venetoclax and 10.15% with 10 μM HMA alone; *p* < 0.001). Conversely, another venetoclax-resistant line, U-937, showed no significant apoptotic response to the combination. In THP-1 cells, this synergy was mediated via a caspase-dependent programmed cell death pathway, evidenced by an increased BAX/BCL-2 ratio, mitochondrial cytochrome c release, and subsequent caspase-9 and caspase-3 activation. Furthermore, co-treatment downregulated the anti-apoptotic protein MCL-1 and reduced PI3K and Akt phosphorylation, suggesting that inhibition of these survival pathways also contributed to the synergistic effect. Inhibition of NHE1 may substantially enhance venetoclax sensitivity in certain AML models, particularly in venetoclax-resistant THP-1 cells but not in U-937, highlighting biological diversity and the probable involvement of alternative survival pathways.

## 1. Introduction

Over the past decade, treatment strategies for acute myeloid leukemia (AML) have undergone substantial transformation, with a growing emphasis on combining targeted agents, such as BCL-2, FLT3, and IDH inhibitors, with conventional chemotherapy to enhance patient outcomes [1,2]. However, despite these advances, substantial improvements in prognosis have not yet been achieved. Many patients continue to experience poor outcomes, characterized by recurrent relapses largely driven by the development of chemoresistance [3]. Consequently, extensive research is ongoing to explore novel combination therapies and other treatment modalities to overcome resistance to these targeted agents.

Na^+^/H^+^ exchanger 1 (NHE1) is a crucial mediator of intracellular pH (pHi) homeostasis in mammalian cells, functioning as a fundamental “housekeeping” protein. It mitigates intracellular acidification by exchanging extracellular sodium ions for intracellular protons [4]. Notably, NHE1 not only maintains pHi in normal cells but also becomes abnormally activated in cancer cells, influencing cancer cell survival, proliferation, and metastasis. Intracellular alkalinization resulting from NHE1 activation has been associated with enhanced cancer cell survival and proliferation, as well as chemotherapeutic resistance, particularly in colon and breast cancer [5,6]. Research on the role of NHE1 in leukemogenesis or chemoresistance in AML is limited, with only a few studies conducted to date. In our previous work, we demonstrated that treatment of leukemic cells with the NHE1 inhibitor 5-(N,N-hexamethylene) amiloride (HMA) reduced their proliferative capacity and induced G1-phase cell cycle arrest [7]. Notably, co-treatment with HMA and cytarabine was effective in overcoming resistance in cytarabine-resistant OCI-AML2 cells as well as in cytarabine-sensitive cells. We also observed that this combination affected the MEK/ERK and PI3K/Akt signaling pathways. While several studies have suggested NHE1 inhibition as a potential approach to overcome chemoresistance in AML [8,9,10], the underlying mechanisms remain unclear, with different mediators identified across studies, highlighting the complex role of NHE1 in chemoresistance.

Venetoclax, a BCL-2 inhibitor, has demonstrated considerable efficacy in treating AML by promoting apoptosis in leukemic cells. BCL-2 is an anti-apoptotic protein that tightly regulates apoptosis initiation by controlling mitochondrial cytochrome c release [11,12]. It prevents mitochondrial membrane permeabilization and apoptosis by sequestering pro-apoptotic proteins such as BAX and BIM. Venetoclax specifically inhibits BCL-2, thereby restoring apoptosis in AML cells. Binding of venetoclax to BCL-2 enables BAX and BIM to promote outer mitochondrial membrane permeabilization. The release of cytochrome c from mitochondria triggers the caspase cascade and downstream apoptotic pathways. Clinical studies have reported notable response rates for venetoclax, particularly when combined with hypomethylating agents or low-dose cytarabine, achieving complete remission in approximately 37–66% and 48–64% of patients, respectively [13,14,15,16]. However, relapse after a short response duration remains a challenge in improving AML survival [13].

One mechanism by which AML cells develop resistance to venetoclax is the upregulation of other anti-apoptotic proteins, including MCL-1, which can sequester BAX and BIM, thus compensating for the loss of BCL-2 function [11,17,18,19]. Additionally, alternative survival pathways—such as PI3K/Akt, MEK/ERK, and RAS/MAPK—have been implicated in venetoclax resistance, as they promote cell survival and proliferation independent of BCL-2 [20,21,22]. Targeting these compensatory mechanisms is therefore a promising strategy for overcoming resistance and improving venetoclax efficacy. Previous studies have shown that combining venetoclax with other targeted inhibitors, such as FLT3 or MCL-1 inhibitors, enhances apoptotic responses in AML cells [11,23,24,25].

Given the functional overlap between NHE1 and BCL-2 family-regulated pathways, we hypothesized that NHE1 may contribute to venetoclax resistance in AML by maintaining pHi and activating survival pathways such as PI3K/Akt. We aimed to determine whether NHE1 inhibition could overcome resistance to venetoclax and enhance its pro-apoptotic effects in AML cells. Furthermore, we sought to elucidate the molecular mechanisms underlying this synergistic effect.

## 2. Materials and Methods

### 2.1. Reagents and Antibodies

All tissue culture media were purchased from Gibco Inc. (Rockville, MD, USA). Dimethyl Sulfoxide (DMSO; tissue culture grade) and HMA were purchased from Sigma-Aldrich (St. Louis, MO, USA). Venetoclax was purchased from Cayman Chemical Co. (Ann Arbor, MI, USA). MK2206 and SC79 were obtained from Selleckchem (Houston, TX, USA). Stock solutions of 5-(N, N-hexamethylene) amiloride (HMA) were prepared by dissolving in DMSO. Working solutions were prepared by diluting in tissue culture medium to achieve the required concentrations, with the final DMSO concentration maintained below 0.1% (*v*/*v*).

Rabbit polyclonal antibodies against caspase-3, caspase-9, poly(ADP-ribose) polymerase (PARP), MCL-1, BIM, BAK, BAX, Akt, phospho-Akt (Ser437), phospho-Akt (Thr308), PI3 kinase, phosphor-PI3 kinase, cytochrome c, and goat anti-rabbit IgG were purchased from Cell Signaling Technology (Danvers, MA, USA). The anti-human NHE1 rabbit polyclonal antibody was purchased from MBL International (Schaumburg, IL, USA).

### 2.2. Leukemia Cell Lines and Treatment

Human leukemia cell lines MV4-11, MOLM-13, RS4;11, THP-1, and U-937 (American Type Culture Collection, Manassas, VA, USA) were cultured in RPMI 1640 medium containing 10% (*v*/*v*) heat-inactivated fetal bovine serum, 100 U/mL penicillin, and 10 µg/mL streptomycin. Cells were maintained at 37 °C in a humidified incubator with 5% CO_2_. For experiments, cells were seeded at a density of 1 × 10^5^ cells/mL into individual wells of a 24-well plate containing 1 mL of RPMI 1640 supplemented with 10% fetal bovine serum and incubated at 37 °C in a humidified atmosphere of 5% CO_2_ and 95% air. Control groups received equivalent volumes of solvent. All assays were performed in triplicate. Cells in the logarithmic growth phase (1 × 10^5^ cells/mL) were treated with either a single agent or combinations of two or more agents, as specified for each experiment.

### 2.3. Annexin-V FITC/Propidium Iodide (PI) Apoptosis Assay

Annexin-V assays were performed according to the manufacturer’s instructions (BD Biosciences, San Jose, CA, USA). Briefly, 2 × 10^5^ cultured cells were plated in 24-well plates, rinsed with Dulbecco’s PBS lacking calcium and magnesium (Cambrex Bioscience, Baltimore, MD, USA), and incubated in 100 µL of a binding buffer containing 5 µL Annexin-V-FITC. Nuclei were counterstained with PI. The proportion of apoptotic cells was quantified using an LSR II or LSRFortessa flow cytometer equipped with FACSDiva software (version 9.0, BD Biosciences).

### 2.4. Western Blot Analysis

After treatment, cells were lysed in buffer containing 50 mM Tris-HCl (pH 7.5), 120 mM NaCl, 20 mM NaF, 1 mM ethylene diamine tetraacetic acid, 5 mM ethylene glycol tetraacetic acid, 15 mM sodium pyrophosphate, 1 mM benzamidine, 0.1 mM phenylmethylsulfonyl fluoride, and 1% Nonidet P-40. The lysates were centrifuged, and the supernatants were collected. Protein concentrations were determined using a detergent-compatible protein assay kit (Bio-Rad, Hercules, CA, USA). Samples were mixed with a sodium dodecyl sulfate (SDS) loading buffer (Beyotime, Shanghai, China) and heated for 5 min. Equal amounts of protein (20 μg) were then subjected to a 10 min heat treatment and separated on a 15% polyacrylamide gel via SDS-polyacrylamide gel electrophoresis. Proteins were subsequently transferred onto nitrocellulose membranes (GE Healthcare, Chicago, IL, USA) via electroblotting. Membranes were blocked with either 5% skim milk or 5% bovine serum albumin, followed by incubation with primary antibodies for 2 h at room temperature. After rinsing, membranes were exposed to the appropriate horseradish peroxidase–conjugated secondary antibody for 1 h. Following four washes with Tris-buffered saline containing Tween 20, target proteins were detected using an enhanced chemiluminescence system (GE Healthcare). To quantitatively evaluate changes in protein expression, densitometric analysis of all Western blot bands was performed using ImageJ software (version 1.53). Band intensities were normalized to β-actin.

### 2.5. siRNA Transfection

NHE1-specific small interfering RNAs (siRNAs) were purchased from Dharmacon (Lafayette, CO, USA). THP-1 cells (2 × 10^6^) were transfected with siRNAs using program U-15 on an Amaxa Nucleofector T device (Lonza Cologne GmbH, Cologne, Germany), following the manufacturer’s guidelines. FITC-labeled non-targeting scrambled siRNA served as the control. The NHE1 siRNA sequence was: 5′-CUGAUGAGUAACAUGAAUA-3′. For transfection, cells were prepared at concentrations of 25 × 10^3^ or 50 × 10^3^ cells per 100 μL of delivery medium. Each reaction contained 1 μM siRNA per 100 μL of delivery medium (3 μg siRNA). Complete culture medium (final FBS concentration of 15%) was then added, and cells were exposed to the indicated concentrations of venetoclax. Cells were harvested for Western blot and apoptosis assays.

### 2.6. Cytosol and Mitochondrial Cytochrome C Assay

Cells were collected, rinsed with ice-cold PBS, and separated into cytosolic and mitochondrial fractions using a commercially available cytosol/mitochondrial isolation kit (Biovision, Inc., Milpitas, CA, USA). Lysates were centrifuged at 600× *g* for 10 min at 4 °C to remove debris, and the supernatant, containing the cytosolic fraction, was collected. The pellet, containing mitochondria, was resuspended in mitochondrial lysis buffer and centrifuged at 12,000× *g* for 15 min at 4 °C to isolate the mitochondrial fraction. Protein concentrations in both the cytosolic and mitochondrial fractions were determined by the Bradford assay to confirm equal loading. Equal amounts of protein from each fraction were separated by SDS–PAGE and subsequently transferred onto PVDF membranes. Western blotting was performed using specific antibodies against cytochrome c to detect its presence in the respective fractions. Detection was performed via enhanced chemiluminescence, and band intensities were compared between cytosolic and mitochondrial fractions.

### 2.7. Statistical Analysis

Data are presented as the mean ± standard error of the mean (SEM) from at least three independent experiments. Comparisons between two groups were performed using a two-tailed Student’s *t*-test in GraphPad Prism version 8.00 for Windows (GraphPad Software Inc., La Jolla, CA, USA). Statistical significance was defined as *p* < 0.05, with the following notations: * *p* < 0.05, ** *p* = 0.01, *** *p* < 0.001, and **** *p* < 0.0001. The combination index (CI) was calculated using the CI equation algorithms implemented in CompuSyn (ComboSyn, Inc., Paramus, NJ, USA). CI values of 1, <1, or >1 indicated additive effects, synergism, or antagonism, respectively [26].

## 3. Results

### 3.1. NHE1 Inhibition with Venetoclax Synergistically Enhances Apoptosis in Venetoclax-Sensitive and -Resistant Leukemic Cell Lines

**Figure 1:**
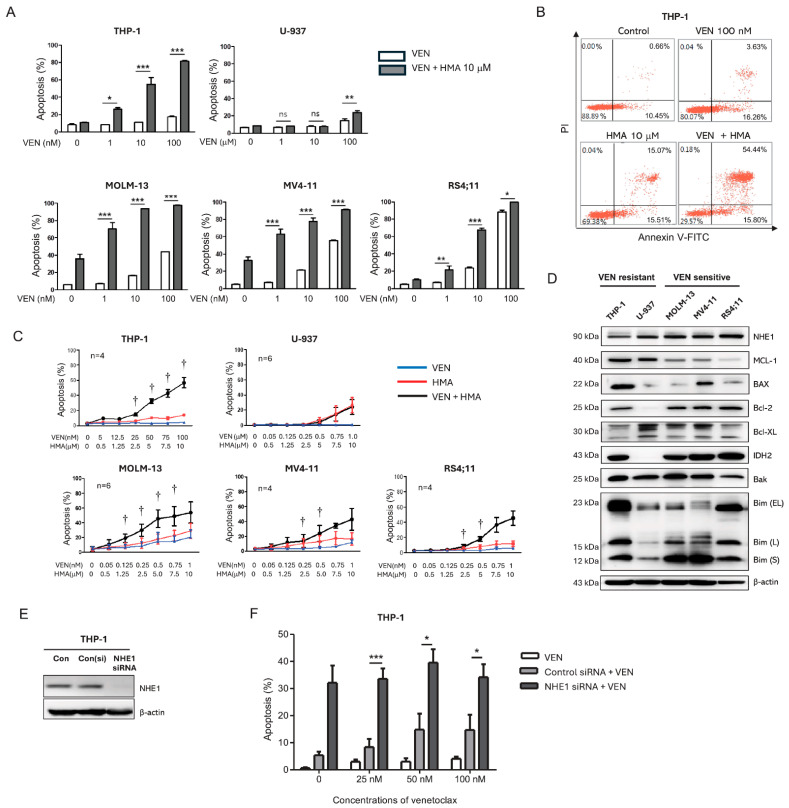
Synergistic induction of apoptosis by venetoclax and Na^+^/H^+^ exchanger 1 inhibition in venetoclax-sensitive and -resistant leukemic cells. (**A**) Apoptotic cell percentages 24 h after co-treatment with the BCL-2 inhibitor (venetoclax) and the NHE1 inhibitor (5-(N,N-hexamethylene) amiloride [HMA]) in five AML cell lines. (**B**) Scatter plot showing apoptosis in THP-1 cells 24 h following treatment with venetoclax (100 nM) and HMA (10 µM). (**C**) Apoptosis rates in THP-1 cells after 24 h treatment with increasing concentrations of venetoclax and HMA at fixed ratios. The combination index was calculated using CompuSyn software. (**D**) Western Blot analysis of NHE1 and BCL-2 family protein expression in venetoclax-resistant cells (THP-1, U-937), and venetoclax-sensitive cells (Molm-13, MV4-11, RS4;11). (**E**) NHE1 knockdown confirmed by Western blot following siRNA transfection in THP-1 cells. (**F**) Apoptotic cell rates in THP-1 cells transfected with 50 nM NHE1 siRNA and treated with venetoclax at the indicated concentrations for 24 h. Bars represent mean ± standard error of the mean (SEM) from ≥3 independent experiments. Statistical analysis was performed using one-way ANOVA followed by Tukey’s multiple-comparison test. ns (non-significant); * *p* < 0.05; ** *p* < 0.01; *** *p* < 0.001; † Combination index < 1. VEN, venetoclax; HMA, 5-(N,N-hexamethylene) amiloride.

To evaluate the pro-apoptotic effects of venetoclax in combination with HMA, five AML cell lines (MOLM-13, MV4-11, RS4;11, THP-1, and U-937) were treated with venetoclax, HMA, or both for 24 h. Venetoclax concentrations (0.1–100 nM) were chosen based on preliminary experiments to achieve approximately 30–50% apoptosis in each cell line. HMA 10 µM alone did not induce significant apoptosis, whereas ≥20 µM caused marked apoptosis in most cell lines, likely due to excessive intracellular acidosis. Therefore, 10 µM HMA was used for subsequent experiments. Venetoclax induced apoptosis in a dose-dependent manner in MOLM-13, MV4-11, and RS4;11 cells, whereas THP-1 and U-937 cells were largely unresponsive (Figure 1A), consistent with their classification as venetoclax-sensitive and -resistant, respectively [27]. In THP-1 cells, which were minimally responsive to either agent alone, the combination treatment markedly increased apoptosis, reaching up to 80% cell death (Figure 1B). To further assess the synergistic effect, the five AML cell lines were treated with venetoclax, HMA, or both for 24 h at fixed combination ratios. HMA alone induced apoptosis in a dose-dependent manner in MOLM-13 and MV4-11 cells but was ineffective in RS4;11, THP-1, and U-937 cells. Notably, the venetoclax–HMA combination significantly enhanced apoptosis in venetoclax-resistant THP-1 cells as well as in all venetoclax-sensitive cell lines (Figure 1C). Synergy was confirmed by CI values < 1 across all tested concentrations, with greater synergy at higher concentrations: 5 nM + 0.5 µM (CI = 0.097), 12.5 nM + 1.25 µM (CI = 0.243), 25 nM + 2.5 µM (CI = 0.126), 50 nM + 5 µM (CI = 0.024), 75 nM + 7.5 µM (CI = 0.012), 100 nM + 10 µM (CI = 0.004). These results confirm a strong synergistic interaction between Venetoclax and HMA in inducing apoptosis, particularly in THP-1 cells. In contrast to THP-1 and venetoclax-sensitive lines, U-937 cells did not undergo apoptosis with the combination. Based on these findings, THP-1 cells were selected for mechanistic studies. Western Blot analysis revealed lower NHE1 expression in venetoclax-resistant THP-1 and U-937 cells, while MCL-1 expression was higher than in venetoclax-sensitive lines. Notably, BAX expression was highest in THP-1 and MV4-11 cells (Figure 1D).

To confirm the role of NHE1 in modulating venetoclax resistance, NHE1 was silenced in THP-1 cells using siRNA (Figure 1E). NHE1 knockdown significantly increased apoptosis compared with scrambled controls. Moreover, treatment of NHE1-knockdown THP-1 cells with venetoclax induced approximately 40% apoptosis, surpassing levels achieved with venetoclax alone (Figure 1F). These results indicate that NHE1 inhibition can sensitize THP-1 cells to venetoclax in this model.

### 3.2. MCL-1 Downregulation Contributes to Synergistic Apoptosis Induced by Venetoclax and HMA

**Figure 2:**
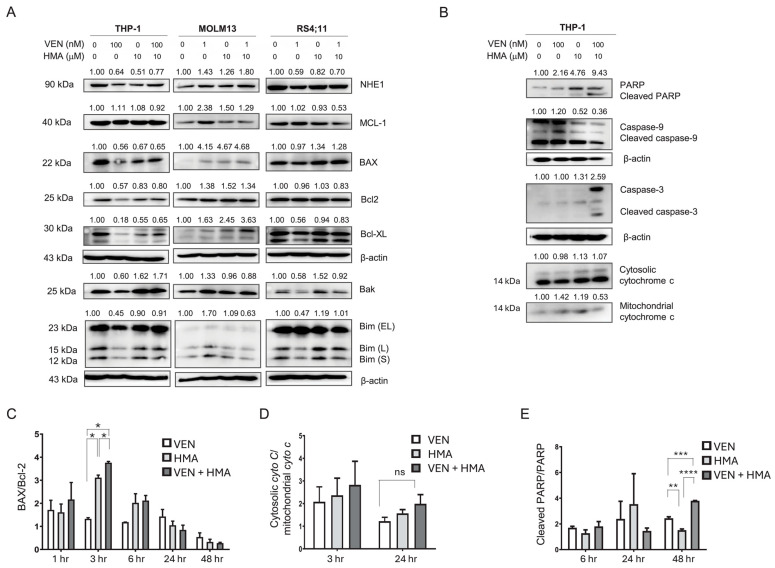
Downregulation of PI3K/Akt signaling and activation of mitochondrial apoptosis by venetoclax and HMA. (**A**) Western Blot analysis of NHE1, MCL-1, BAX, BCL-2, BCL-XL, and BIM isoforms (EL, L, S) in THP-1, MOLM-13, and RS4;11 cells after 24 h treatment with venetoclax, HMA, or both. (**B**) Western Blot showing levels of caspase-9, caspase-3, PARP, and cytosolic/mitochondrial cytochrome c in THP-1 cells treated for 24 h. (**C**–**E**) Bar graphs quantifying the BAX/BCL-2 ratio, cleaved PARP/total PARP ratio, and cytosolic/mitochondrial cytochrome c ratio in THP-1 cells. Measurements were performed at 3, 6, 24, or 48 h, depending on the target protein, as indicated in the graphs. For each time point, protein ratios in the control group were normalized to 1 and used as the reference for calculating relative ratios. Bars represent mean ± SEM from ≥3 independent experiments. Statistical comparisons were analyzed using one-way ANOVA with Tukey’s post hoc test, and significance is indicated as ns (non-significant), * *p* < 0.05; ** *p* < 0.01; *** *p* < 0.001; **** *p* < 0.0001. VEN, venetoclax; HMA, 5-(N,N-hexamethylene) amiloride; Cyto C, cytochrome C, ns, non-significant.

To elucidate the molecular basis of the synergistic effect, we examined the changes in the expression of BCL-2 family proteins, including MCL-1, BCL-2, BCL-XL, and BAX, following treatment. Baseline MCL-1 expression, determined by Western Blot, was elevated in THP-1 cells compared with MOLM-13 and RS4;11 cells (Figure 2A). Combination treatment with 100 nM venetoclax and 10 µM HMA significantly downregulated MCL-1 expression in THP-1, MOLM-13, and RS4;11 cells, correlating with increased apoptosis.

MCL-1, a member of the BCL-2 family with anti-apoptotic functions, is a key contributor to chemoresistance in multiple cancers, including leukemia [28]. Overexpression of MCL-1 promotes leukemic cell survival by binding to and neutralizing pro-apoptotic proteins such as BAX and BAK [29]. Our findings, together with previous reports, suggest that elevated MCL-1 levels contribute to venetoclax resistance in THP-1 cells and that this resistance can be overcome by NHE1 inhibition.

### 3.3. Activation of Intrinsic Apoptosis via Mitochondrial Dysfunction and Caspase Signaling Also Contributes to the Synergistically Induced Apoptosis by Venetoclax and HMA

To further clarify the mechanisms underlying apoptosis induced by combination therapy, we analyzed key apoptotic signaling events at 1, 3, 6, 24, and 48 h after treatment. Although changes in signaling molecules associated with intrinsic apoptosis were observed at 24 h (Figure 2B), upregulation of BAX and an elevated BAX/BCL-2 ratio were detectable as early as 3 h post-treatment, particularly in the HMA and combination groups, indicating the early activation of pro-apoptotic signaling (Figure 2C). This shift reflects increased permeabilization of the mitochondrial outer membrane, a hallmark of intrinsic apoptosis.

At both 3 and 24 h, translocation of mitochondrial cytochrome c into the cytosol was observed in the combination group, indicating mitochondrial damage (Figure 2D). Furthermore, increased levels of cleaved caspase-9, caspase-3, and PARP were detected at 24 and 48 h post-treatment, confirming initiation of the caspase cascade and the progression of apoptosis (Figure 2B,E). Cleavage of caspase-9, an essential initiator caspase, and caspase-3, an executioner caspase, confirmed involvement of the intrinsic apoptotic cascade. The increase in PARP cleavage, a substrate of caspase-3, further supports activation of intrinsic caspase-dependent apoptosis. Together, these results indicate that venetoclax plus HMA enhances apoptosis by inducing mitochondrial dysfunction and activating the intrinsic apoptotic pathway.

### 3.4. Inhibition of the PI3K/Akt Pathway Mediates Reversal of Venetoclax Resistance by NHE1 Inhibition

**Figure 3:**
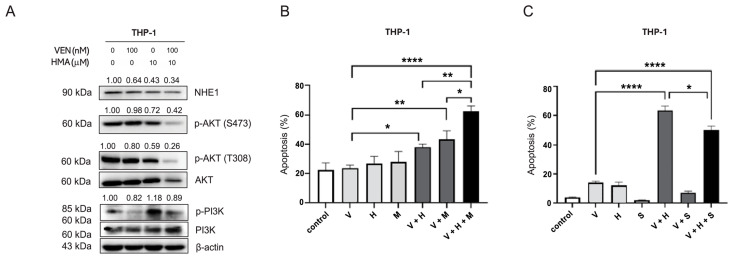
Modulation of Akt activity alters the apoptotic response to combined venetoclax and NHE1 inhibition. (**A**) Western Blot analysis of phosphorylated PI3K/Akt, MCL-1, BCL-2 family proteins, cleaved caspase-9, and PARP in THP-1 cells following 24 h treatment with venetoclax (100 nM), HMA (10 µM), or both. (**B**) Apoptosis rates after 24 h of treatment with venetoclax (100 nM), the NHE1 inhibitor HMA (10 µM), and/or the Akt inhibitor MK2206 (1 µM). (**C**) Apoptosis rates following 24 h treatment with venetoclax (100 nM), HMA (10 µM), and/or the Akt activator SC79 (1 µM). V: venetoclax (100 nM); H: HMA (10 µM); M: MK2206 (1 µM); S: SC79 (1 µM). Bars represent mean ± SEM from ≥3 independent experiments. Statistical analysis was performed using one-way ANOVA followed by Tukey’s multiple-comparison test. * *p* < 0.05; ** *p* < 0.01; **** *p* < 0.0001.

The elevated MCL-1 expression observed in THP-1 cells and its downregulation following HMA treatment suggest that upstream survival pathways, including PI3K/Akt and MAPK/ERK, mediate venetoclax resistance. To explore this, we assessed kinase activity by Western Blot in THP-1 cells treated with venetoclax, HMA, or both. After 24 h of treatment, Akt phosphorylation was markedly reduced in the combination group, correlating with increased apoptosis (Figure 3A). Phosphorylated PI3K levels were also significantly decreased by combination treatment compared with either agent alone, supporting enhanced inhibition of the PI3K/Akt pathway. Given the pivotal role of the PI3K/Akt cascade in cell survival by phosphorylating and inhibiting pro-apoptotic proteins such as Bad and caspase-9, its inactivation is likely to facilitate apoptosis in venetoclax-resistant cells.

To directly assess the role of Akt in mediating resistance, we pharmacologically modulated its activity. Treatment of THP-1 cells with the Akt inhibitor MK2206 (2.5 µM) significantly enhanced venetoclax-induced apoptosis compared with venetoclax alone (V: 23.6% vs. V+M: 43.5%, *p* < 0.01) and further increased apoptosis when combined with HMA (V+H: 37.9% vs. V+H+M: 62.17%, *p* < 0.05) (Figure 3B). Conversely, co-treatment with the Akt activator SC79 (1 µM) partially reduced apoptosis induced by the venetoclax–HMA combination (V+H: 63.5% vs. V+H+S: 50.2%, *p* < 0.05) (Figure 3C). These findings demonstrate that Akt inhibition enhances, whereas Akt activation diminishes, the apoptotic effects of venetoclax–HMA co-treatment, indicating that suppression of Akt signaling facilitates venetoclax sensitization in resistant AML cells.

## 4. Discussion

This study shows that inhibition of NHE1 with HMA can synergistically induce apoptosis in venetoclax-sensitive leukemic cells by enhancing their response to venetoclax, and in venetoclax-resistant THP-1 cells. The robust synergistic apoptotic response elicited by co-administration of venetoclax and HMA was mediated through mitochondrial dysfunction and activation of the intrinsic caspase-dependent cell death pathway. Notably, our results showed that downregulation of MCL-1 and decreased Akt phosphorylation were associated with this synergy, suggesting that these pathways contribute to venetoclax resistance and are reversed by NHE1 inhibition. However, the mechanistic findings described here were most prominent in THP-1 cells; therefore, while NHE1 modulation may overcome venetoclax resistance in certain AML subsets, these results should not be generalized to all forms of AML.

NHE1 is known to promote chemoresistance by increasing intracellular pH and activating prosurvival pathways, such as PI3K/Akt and MEK/ERK, which enhance the expression of anti-apoptotic proteins, including MCL-1. Our previous studies demonstrated that NHE1 inhibition with HMA sensitizes cytarabine-resistant OCI-AML2 cells to chemotherapy, and this sensitization is accompanied by decreased Akt phosphorylation [7]. In the present study, the synergistic effect of HMA and venetoclax in venetoclax-resistant THP-1 cells was likewise associated with reduced phosphorylation of the PI3K/Akt pathway, suggesting a potential underlying mechanism. The downregulation of Akt phosphorylation observed here is consistent with our previous findings in cytarabine-resistant cells. Given that the PI3K/Akt/mTOR pathway is a well-recognized regulator of leukemic cell survival and chemoresistance [30,31,32,33], our results suggest that NHE1 activation may confer resistance to both venetoclax and cytarabine by upregulating anti-apoptotic signaling and suppressing caspase-9 and PARP activation.

Our results indicate that NHE1 activity, rather than its total expression, may drive venetoclax resistance in certain AML subsets, explaining why resistant cells remain responsive to NHE1 inhibition despite lower NHE1 protein levels. Functional activation of NHE1 is likely regulated by post-translational modifications, altered localization, or upstream kinase signaling, rather than solely by protein abundance. This interpretation is supported by previous reports that NHE1 hyperactivation maintains intracellular alkalinity and prevents mitochondrial apoptosis, thereby promoting leukemic-cell survival. A notable observation in this study is the heterogeneity of AML cell responses to venetoclax–HMA co-treatment: while THP-1 cells exhibited pronounced synergy, U-937 cells were unresponsive, indicating that NHE1 activation is not a universal driver of venetoclax resistance. Such diversity likely reflects differential reliance on alternative survival pathways—including PI3K/Akt, MAPK, NF-κB, and lysosome-mediated stress responses—that compensate for the loss of NHE1-mediated homeostasis. Thus, NHE1 targeting can enhance venetoclax sensitivity in select AML models, but its efficacy is context-dependent and influenced by cell-specific signaling profiles.

Consistent with these findings, previous studies have demonstrated that NHE1 activation or overexpression contributes to therapy resistance in AML. Man et al. demonstrated that NHE1 inhibition via co-treatment with sorafenib and HMA reversed sorafenib resistance in FLT3-ITD+ AML cells, accompanied by inhibition of FLT3 signaling and the phosphorylation of STAT5, Akt, and ERK [10]. They also reported that NHE1 is phosphorylated in a kinase-dependent manner—such as by FLT3-ITD, KRASG12D, or BTK—which maintains an alkaline intracellular pH in AML cells and promotes leukemia cell survival [9]. Furthermore, simultaneous inhibition of kinases and NHE1 synergistically reduced intracellular pH and suppressed AML growth, whereas combining kinase inhibitors, such as quizartinib or ibrutinib, with amiloride enhanced intracellular acidification and cytotoxicity. These results collectively support our interpretation that functional hyperactivation—rather than overexpression—of NHE1 contributes to venetoclax resistance and explains the sensitivity of THP-1 cells to HMA despite their relatively low baseline NHE1 expression.

Importantly, the synergistic cell death observed with venetoclax and HMA does not appear to result from nonspecific cytotoxicity. In our preliminary experiments, HMA concentrations ≥ 20 µM induced marked apoptosis in most AML cell lines, likely due to excessive intracellular acidosis; therefore, subsequent experiments used 10 µM or lower, which showed minimal cytotoxicity when applied alone. Under these optimized conditions, the combination selectively activated the intrinsic apoptotic pathway by downregulating MCL-1, inducing a BAX/BCL-2 imbalance, releasing cytochrome c, and activating caspases, indicating a pathway-specific rather than nonspecific toxic effect.

MCL-1, a member of the BCL-2 protein family, is a well-established anti-apoptotic protein whose overexpression has been implicated in venetoclax resistance. Several studies have shown that targeting MCL-1 can restore sensitivity to venetoclax. Overexpression of MCL-1 may result from genetic alterations, such as gene amplification, chromosomal translocations, or mutations, as well as from activation of survival-promoting signaling cascades such as PI3K/Akt and MEK/ERK. Rahmani et al. [34] emphasized the role of PI3K/Akt-driven MCL-1 upregulation in AML chemoresistance, showing that treatment with venetoclax and the PI3K inhibitor GDC-0980 rapidly promoted BAX translocation, cytochrome c release from mitochondria, and apoptosis across diverse AML cell lines, along with inhibition of Akt/mTOR activity and decreased MCL-1 expression. In line with these findings, our results suggest that activation of the PI3K/Akt pathway contributes to venetoclax resistance by sustaining MCL-1 expression, and that this resistance can be reversed through NHE1 inhibition.

This study has several limitations. We did not conduct a comprehensive screening across multiple AML models, and additional validation using primary AML cells or In Vivo experiments would strengthen our findings. Further molecular characterization—including kinase mutation and BH3 profiling—will be required to clarify the mechanisms underlying the enhanced venetoclax sensitivity observed with NHE1 inhibition. The absence of response in U-937 highlights the heterogeneity of venetoclax resistance and the need to define non-responder subgroups. Moreover, the lack of negative-control cell types, such as normal hematopoietic progenitors, limits direct assessment of specificity; future work will incorporate these models to confirm the selectivity of NHE1-targeted modulation. Despite these limitations, our data support NHE1 inhibition as a promising strategy to overcome chemoresistance in AML.

## 5. Conclusions

In conclusion, inhibition of NHE1 enhances leukemic cell sensitivity to venetoclax, producing a synergistic pro-apoptotic effect mediated by MCL-1 downregulation and Akt inhibition. These findings suggest that NHE1 modulation can restore BCL-2 inhibitor sensitivity by targeting key survival pathways in specific AML contexts. Further studies are warranted to identify predictive biomarkers and validate their therapeutic potential in preclinical and clinical models.

## Data Availability

The datasets generated and/or analyzed during the current study are available from the corresponding author upon reasonable request.

## Abbreviations

The following abbreviations are used in this manuscript:

AML	Acute Myeloid Leukemia
BCL-2	B-cell lymphoma 2
DMSO	Dimethyl Sulfoxide
FBS	Fetal Bovine Serum
H^+^	Proton (Hydrogen ion)
IC_50_	Half Maximal Inhibitory Concentration
IDH	Isocitrate Dehydrogenase
MCL-1	Myeloid Cell Leukemia 1
Na^+^	Sodium ion
NHE1	Na^+^/H^+^ Exchanger 1
PBS	Phosphate-Buffered Saline
ROS	Reactive Oxygen Species
RPMI	Roswell Park Memorial Institute (medium)
RT-qPCR	Reverse Transcription Quantitative Polymerase Chain Reaction
SD	Standard Deviation
SEM	Standard Error of the Mean
WB	Western Blot
